# The Sense and Sensibility of Strand Exchange in Recombination Homeostasis

**DOI:** 10.1371/journal.pgen.1004104

**Published:** 2014-01-23

**Authors:** Francesca Cole

**Affiliations:** Department of Molecular Carcinogenesis, University of Texas MD Anderson Cancer Center, Smithville, Texas, United States of America; National Cancer Institute, United States of America

Upon entering a Regency-era ball, a Jane Austen heroine might ask herself, “Do I stay with my sister, or attempt to secure a partner?” The homologous recombination events that occur during meiosis need to make a similar decision, and how they do so is investigated in papers by Doug Bishop, Neil Hunter, and colleagues [1] and Nancy Hollingsworth and colleagues [2] in *PLOS Genetics*. The authors define the interplay between the strand exchange proteins Dmc1 and Rad51 in partner choice during meiotic double-strand break (DSB) repair and reveal that when the delicate balance between their activities is manipulated, partner choice is disrupted. However, like the plot twists that bring partners together to enforce happy endings in Austen's novels, robust buffering of recombination counteracts the deleterious effect of eschewing a partner in favor of your sister (chromatid).

For many organisms, accurate segregation of homologous chromosomes of different parental origin (homologs) in meiosis depends upon interhomolog crossovers. Crossovers are formed by the repair of programmed DSBs induced by Spo11 [3] and involve the exchange of genetic information flanking the initiating break. DSB ends are processed into 3′ single-stranded tails encased by a nucleoprotein filament, which contains strand exchange factors that are used to invade an intact homologous duplex to initiate recombination. Recombination intermediates can be resolved to form crossovers or noncrossovers, the latter of which do not involve exchange of flanking information. Failure to form crossovers leads to aneuploidy or gamete death; as such, crossover frequency and distribution is tightly controlled to ensure at least one crossover occurs between each pair of homologs (crossover assurance), that crossovers do not form in close juxtaposition (crossover interference), and that crossover numbers are maintained despite perturbations in the number of DSBs (crossover homeostasis) [4]. Recent studies of crossover control have revealed feedback regulation occurring at multiple steps during recombination progression—including DSB formation [5]–[7] and the crossover-noncrossover decision [8]—to provide robust homeostatic control. Critical questions remain: what intermediates are sensed by homeostatic mechanisms, and how do these mechanisms interact to execute crossover regulation?

Like DSB formation, the strand exchange reaction is a potential target of homeostatic control. The Rad51 strand exchange factor mediates recombination during the mitotic cell cycle, which preferentially utilizes the sister chromatid to template repair. While Rad51 also has a critical role in meiosis, its own strand exchange activity is not required. Instead, Rad51 functions as a cofactor for a meiosis-specific strand exchange factor, Dmc1 [9]. When Rad51 and Dmc1 act together, they exhibit homolog bias, directing strand exchange between homologs rather than sister chromatids. However, loss of Rad51 does not phenocopy loss of Dmc1 [3]. In the absence of Rad51, interhomolog recombination is reduced, and intersister recombination predominates. In the absence of Dmc1, all DSB repair is dramatically reduced, a recombination checkpoint is activated, and the strand exchange activity of Rad51 is inhibited by the Hed1 protein and by an effector kinase, Mek1. Removing this inhibition allows efficient recombination, although interhomolog crossovers are reduced compared to wild type.

Lao, Cloud, et al. [1] asked how well budding yeast *dmc1 hed1* mutants, which use Rad51 alone, complete meiosis. Using assays that differentiate template choice in recombination intermediates, they observed a five-fold reduction in homolog bias in *dmc1 hed1* and a two-fold reduction in *hed1* alone. This implies that Dmc1 executes template choice by inhibiting the strand exchange activity of Rad51. In a complementary study, Liu et al. [2] took a different approach by generating a hypomorphic allele, *dmc1-T159A*. They then tweaked the balance of power between Rad51 and Dmc1 by introducing *dmc1-T159A* into strains lacking Hed1 and/or a bypass of Mek1 repression of Rad51. While *dmc1-T159A* showed no reduction in homolog bias on its own, coupling it with increased Rad51 activity led to a synergistic eight-fold reduction in homolog bias, inferring that inhibition of Rad51 strand exchange is required to favor interhomolog strand exchange by Dmc1.

Intriguingly, regardless of the extent of reduction in homolog bias, there was a much milder effect on ultimate crossover formation. Lao, Cloud, et al. found that *dmc1 hed1* exhibited nearly wild-type crossover levels and distributions on chromosome III, suggesting that a highly effective compensatory mechanism is invoked. One such mechanism could be the maintenance of crossovers at the expense of noncrossovers [8]; indeed, the authors observed an increase in the crossover-to-noncrossover ratio in *dmc1 hed1*. To test the extent of this compensation, they coupled *dmc1 hed1* with *spo11* alleles that reduce global DSB numbers. Decreasing DSBs did not cause a further increase in the crossover-to-noncrossover ratio, implying that the *dmc1 hed1* strain has maximized the buffering capacity afforded by the crossover-noncrossover decision. While this analysis by Lao, Cloud, et al. was limited to a single recombination hotspot, Liu et al. directly measured genome-wide recombination by whole-genome sequencing of tetrads from strains with altered interhomolog bias. Although crossover number in *dmc1-T159A* was equivalent to wild type, noncrossovers were significantly reduced. In *hed1* alone or in *hed1 dmc1-T159A* double mutants, crossovers were significantly reduced, but noncrossovers were reduced to the same extent as that observed in *dmc1-T159A* alone. Despite the fact that the number of sequenced tetrads was low, these findings indicate that any compensatory mechanisms that work on the crossover-noncrossover decision are already maximized by the mild reduction in interhomolog bias observed in *hed1*. Remarkably, the spacing of crossovers was unaffected in all three strains, indicating that reduced interhomolog interactions do not alter crossover interference.

**Figure 1:**
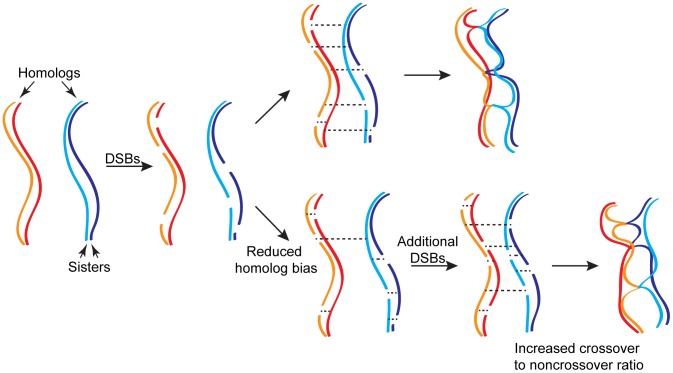
Homeostatic regulation of meiotic recombination. Wild-type meiosis, which has a marked preference for interhomolog recombination, is shown at the top. Dashed lines indicate recombination-dependent interactions that can occur either between sisters or homologs. A proportion of interhomolog interactions will become crossovers that exchange flanking genetic information between homologs and are required for accurate chromosome segregation. The remaining interhomolog interactions are likely to become noncrossovers, which constitute a patch-like repair at the site of the break and do not exchange flanking markers. Aberrant meiosis with reduced homolog bias is shown at the bottom. Additional DNA double-strand breaks (DSBs) are proposed to form in response to reduced interhomolog interactions. Further, when the frequency of successful interhomolog interactions is reduced, the ratio of crossover to noncrossover outcomes is increased. Together, the two types of homeostatic regulation work to maintain crossover number and distribution.

What mechanisms maintain crossover numbers genome-wide? Lao, Cloud, et al. argue that a second homeostatic mechanism—continued formation of DSBs—must account for the high levels of crossovers observed when interhomolog bias is disrupted (Figure 1). In support of this view, while crossovers were reduced locally in *dmc1 hed1* at a hotspot that is saturated for DSB formation, crossovers increased in a larger adjacent chromosomal region. They propose that larger chromosomal regions retain a higher potential for receiving additional DSBs through this second homeostatic mechanism.

An important insight gleaned from these studies is that successful interhomolog interactions are a critical gauge by which homeostatic regulation is effected. Thus, homeostatic sensing must occur between chromosomes (*in trans*) in response to interhomolog interactions, which may or may not be independent from sensing along a chromosome (*in cis*) in response to DSB formation [7]. Recent evidence of multiple types of feedback regulation of Spo11-mediated DSB formation will likely provide a key component to a synthetic model that can explain local, regional, and global regulation of meiotic recombination [5]–[7]. It is notable in this context that the mutants analyzed in the current studies show a delay in meiotic progression, suggesting that they activate the meiotic recombination checkpoint. One recently identified pathway for Spo11-mediated DSB feedback involves the recombination checkpoint preventing expression of proteins that shut off DSB formation [10], [11]. In the template choice mutants studied here, the prophase delay could lead to increased formation of DSBs, which eventually provoke enough interhomolog interactions to disengage the checkpoint and complete meiosis. Recent work in mouse spermatocytes indicates that DSB formation is significantly increased when chromosomes have failed to synapse in this organism, as well [12]. Taken together, these findings in yeast and in mice suggest the existence of highly tuned compensatory mechanisms able to target de novo DSB formation specifically to regions of the genome where more recombination is needed.

These two manuscripts have clarified the competitive relationship between Rad51 and Dmc1 during meiotic strand exchange and leveraged this relationship to investigate how interhomolog interactions are integrated into homeostatic regulation. Identifying all of the various components of the multilayered feedback mechanisms inherent to recombination homeostasis, and especially understanding how they crosstalk with one another, will continue to be a stimulating area of research.
